# A global bibliometric analysis of *Plesiomonas*-related research (1990 – 2017)

**DOI:** 10.1371/journal.pone.0207655

**Published:** 2018-11-29

**Authors:** Temitope Cyrus Ekundayo, Anthony I. Okoh

**Affiliations:** 1 SAMRC Microbial Water Quality Monitoring Centre, University of Fort Hare, Alice, Eastern Cape, South Africa; 2 Applied and Environmental Microbiology Research Group, Department of Biochemistry and Microbiology, University of Fort Hare, Alice, Eastern Cape, South Africa; 3 Department of Biological Sciences, University of Medical Sciences, Ondo City, Ondo State, Nigeria; American University of Beirut, LEBANON

## Abstract

*Plesiomonas shigelloides* is an emerging pathogen with damaging effects on human health such as gastroenteritis and extraintestinal infections. Here, we carried out a bibliometric survey that aimed to examine publication trends in *Plesiomonas*-related research by time and place, international collaborative works, identify gaps and suggest directions for future research. The search term “*Plesiomonas shigelloides*” was used to retrieve articles published between 1990 and 2017 from the Web of Science database. Only primary research articles were included in the analysis. A total of 155 articles were published within the survey period, with an average of 5.54±2.66 articles per year and an annual growth rate of −0.8%. Research output peaked in 2000 and 2006 (each accounting for 7.7% of the total). The United States ranked first in terms of numbers of articles (n = 29, 18.1%) and total citations (n = 451). Cameroon, Canada, Cuba, Switzerland and Turkey co-shared the 10^th^ position each with 2 articles (1.3%). Research collaboration was low (collaboration index = 3. 32). In addition to *Plesiomonas shigelloides* (n = 82, 52.9%), the top Authors Keywords and research focus included lipopolysaccharide and nuclear magnetic resonance (n = 13, 8.4%). Diarrhea (n = 43, 27.7%), *Aeromonas* species (n = 41, 26.5%) and infections (n = 31, 20.0%) were also highly represented in Keywords-Plus. Authors’ collaborations and coupling networks formed two mega-clusters which nodes were shared solely by authors from high-income countries. The common conceptual framework in retrieved articles determined by K-means clustering revealed three clusters with sizes of 7, 16, and 29, representing research responses focused on extraintestinal and gastroenteritis, *P*. *shigelloides* lipopolysaccharide structure, and co-infections, respectively. Our bibliometric analysis revealed a global diminishing research in *Plesiomonas*; greater research outcomes from high-income countries compared to others and low collaboration with developing countries.

## Introduction

*Plesiomonas shigelloides* is a bacterium that has been labeled as an emerging pathogen for over three decades. There are many outstanding questions regarding its pathogenic potential, despite evidence for its detrimental effects on human health such as gastroenteritis and extraintestinal diseases [1–7]. Also, some food and waterborne outbreaks have been traced to *P*. *shigelloides* [8–13], and the incidence of *Plesiomonas* infections linked to immunocompromised health [14] is increasing, especially in light of present-day lifestyles [15]. Climate change and global warming are also predicted to contribute to increased incidence of waterborne infectious diseases including *Plesiomonas* infections [16–19].

Accurate estimates of the incidence of *Plesiomonas*-related gastroenteritis and extraintestinal infections both globally and at the level of individual countries remain unknown [1,2,4–7,10,20–37]. Retrospective reviews of infections due to *P*. *shigelloides* in China and Hong Kong have been published [38], and cases of *P*. *shigelloides* co-infection with viral and bacterial diarrheal pathogens are common in the literature [39]. Prevalence of *P*. *shigelloides* gastroenteritis varies considerably across regions, with lower rates reported from North America and Europe and higher estimates from Southeast Asia and Africa [40]. Nonetheless, there is a general underestimation of *P*. *shigelloides* infection, in part because it shares some clinical manifestations with other pathogens [38]. *P*. *shigelloides* is not routinely examined in clinical settings, and as such, awareness regarding this pathogen remains limited.

Bibliometric analysis is a statistical method for assessing both the quantitative and qualitative scope and adequacy of research efforts attained in an area of interest [41]. It can be used to determine national and international research focus and evaluate research performance in order to identify future research priorities, funding sources, and interdisciplinary collaborations [42–44]. It also provides a resource to policy-makers for implementing necessary prophylactic measures in case the analysis reveals a sharp increase in case reports or articles regarding a health issue in a particular geographic area [45,46]. Bibliometric reviews can additionally help international health agencies to identify priorities (e.g. by nations) for disbursing aid [47] and awarding research grants.

There have been a few recent reviews on *P*. *shigelloides* [2,14,48] but no comprehensive surveys of published studies on *P*. *shigelloides* have been yet conducted. On the other hand, bibliometric analyses have been applied to global disease research on viral agents such as dengue virus [49], Ebola virus [50], John Cunningham virus [51], Mayaro virus [52], Middle East respiratory syndrome coronavirus [53][34], yellow fever virus [54], West Nile virus [55], and Zika virus [56]; and bacterial agents such as *Campylobacter* [45], *Leishmania* species [57], and *Mycobacterium tuberculosis* [58]. Other bibliometric analyses have addressed *Plasmodium* species and resistant malaria vectors [59,60], *Toxocara* species [43], and antifungal triazole resistance (especially in *Candida* and *Aspergillus* species) [46].

Here we carried out a bibliometric analysis of studies on *P*. *shigelloides* published between 1990 to 2017. The articles were evaluated in terms of annual and country-specific output, theme, domain clusters, international collaboration networks, citations, topical evolution related to keywords and co-occurrence networks, co-authorship, and funding. The aim of the survey was to evaluate international participation in *P*. *shigelloides* research—with a special interest in regions where *Plesiomonas* infections have higher prevalence rates (i.e., Africa and Southeast Asia)—in order to address knowledge gaps and provide a resource that can help identify present and future research priorities.

## Methods

### Preamble and terms definition

Bibliometrix package is a suite of tools for accurate publication data processing such as file conversion, term extraction, duplicate matching and merging, descriptive analysis, matrix building and similarity normalization for network analysis [61]. Matrices are built from publication dataset (e.g.; authors, words, countries, references, keywords) for coupling, co-citation, collaboration, conceptual framework and multiple correspondence analyses. Bibliographic coupling occurs between two articles ⅈ and ⅉ when their reference lists cited at least one common source [62]. But, in a collaboration network, the nodes comprise authors and the links co-authorships [63]. The number of bibliographic coupling that occurs between articles ⅈ and ⅉ or co-authorship in scientific collaboration network denotes the strength of the network [61]. A network depicts relationships in a system as a set of nodes (components) and links (relationships) [64]. Co-Word or conceptual framework analysis explore K-means clustering and other dimensionality reduction techniques to identify clusters of common concepts known in a bibliographic collection. It relies on word co-occurrences in a publication dataset [61,64]. Scientific productivity or an author’s contributions in a field is evaluated in term of Lotka’s law [65]. The Lotka’s law is an inverse square law that describes how often authors published in a field [65].

### Data retrieval

Published peer-reviewed articles on *P*. *shigelloides* were retrieved from the Web of Science (WoS) database on August 19, 2018. The WoS is among the most reliable and comprehensive databases for bibliometric studies and hosts a wide range of quality and high-impact scientific studies (12 million articles in over 12,000 journals) [44]. We used the search term “*Plesiomonas shigelloides*” to identify primary research articles published between 1990 and 2017. All available information was retrieved. To obtain subject-specific results and for the sake of accuracy (in order to avoid false-positive results), only article titles were searched. A title-specific search has been reported to increase recovery and specificity with a minimal loss of sensitivity compared to a topic search [44,45,66]. Articles were downloaded in the BibTeX file format. In order to account for variations in country population on rate of scientific production, the world population was retrieved from the World Bank website (https://data.worldbank.org/indicator/SP.POP.TOTL) and the mid-period population corresponding to the top 19 countries was extracted for calculation of article per million populations. England population data was retrieved from Office for National Statistics website (https://www.ons.gov.uk/peoplepopulationandcommunity/populationandmigration/populationestimates/timeseries/enpop/pop).

### Data processing and analysis

We analyzed the retrieved data for bibliometric indicators using Rstudio v.3.4.1 software (2017-06-30) with bibliometrix R-package (http://www.bibliometrix.org) [61]. Data were imported into RStudio and converted to a bibliographic data frame and normalized for duplicate marching. The duplicated article was reduced to one record in the analysis. The data frame as typical columns named after the standard ISI WoS Field Tag codify.

Further, authors' names, authors' keywords (DE), and Keywords-Plus (ID) were extracted for standardization. Authors' names were extracted twice as two different sets (A and B). Each set was checked for variant names, spelling errors and matched with affiliations. We achieved normalized authors' names when |*A* ∩ *B*| ≡ |*A* ∪ *B*|. For keywords (DE) and Keywords-Plus (ID), a primary term was assigned to words with similar meanings (e.g., “*Plesiomonas shigelloides*", "Plesiomonas", "shigelloides", "Aeromonas-shigelloides", and "Plesiomonas-shigelloides" were allotted to “*Plesiomonas shigelloides*”). Multiple occurrences of a keyword or a similar keyword in an article were regarded as one. Co-occurrence of a term in authors' keywords (DE set) and Keywords-Plus (ID set) in the dataset was assessed as a set made of the intersect of the two sets (*DE* ⋂ *ID*||). All set-based test was performed using a Venn diagram software (http://bioinformatics.psb.ugent.be/webtools/Venn/). An annual number of articles and total citations were also graphed.

Data were analysed for descriptive output, citation analysis, authors’ h-index and scientific productivity using the relevant functions of the bibliometrix R-package. Bibliometric networks (e.g., citation, author, country, author keyword, and Keywords-Plus networks) and bibliographic coupling (co-citation and keyword co-occurrences) were computed and visualized from bibliometric two-way (bipartite) network of rectangular matrices of Articles × Attributes. A typical bibliometric network is expressed as *Network*(*N*) = *X* × *N^T^* where *X* is a bipartite network matrix composed of Articles × Attribute (e.g. Authors, keywords, citations, and Countries) and *N* is a symmetrical matrix N = N^T^.

We created a graphic model of all networks using force-directed algorithms (Fruchterman) implemented in the networkPlot function of the bibliometrix R-package. All networks were standardized using the Simpson’s coefficient (inclusion index), proximity index (association strength), the Jaccard’s similarity index, and the Salton’s cosine coefficient among nodes of a network [61]. In addition, k-means clustering were performed on keywords to evaluate concepts in *Plesiomonas* field of research using the function conceptualStructure of the package. The function implements Porter’s stemming algorithm [67] to modulate inflected words to their root form. For detailed search Boolean for articles identification from WoS, see supplementary file (S1 Appendix). Other bibliometric indicators such as language and affiliation were determined using the content search of BibTeX file.

## Results

**Fig 1:**
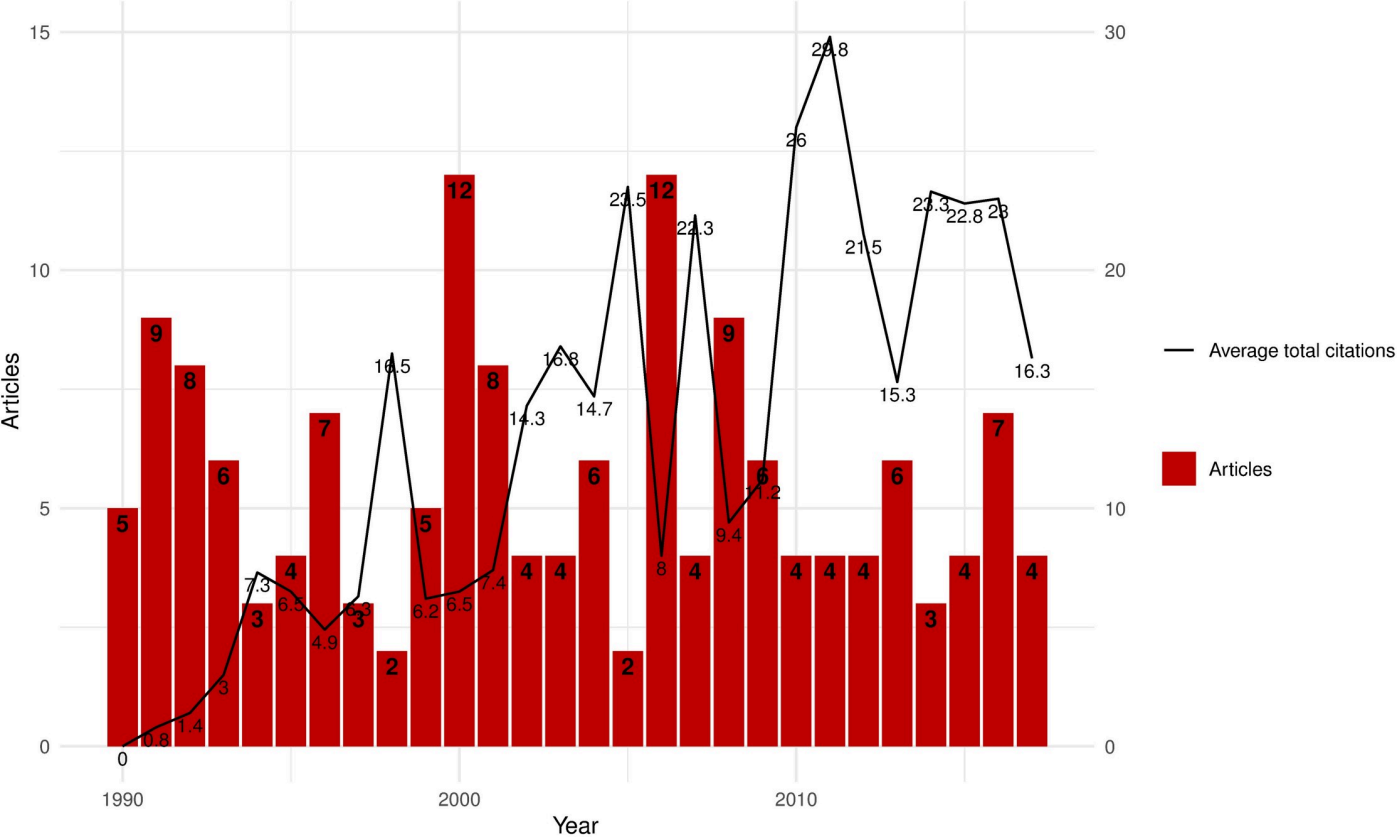
Published studies on *P*. *shigelloides* from 1990 to 2017. ATC, average total citations of articles published in a year. The annual growth rate was −0.8%. Research output fluctuated during the study period and peaked in 2000 and 2006 (12 articles [7.7%] in each of those years; mean: 5.54±2.66 per year, range: 2.0–12.0).

A total of 155 articles were published within the survey period; their attributes are presented in Table 1. The studies involved 493 authors, with 0.31 article/author (3.18 authors/article), 4.34 co-authors/article, and a collaboration index of 3.32. With the exception of two authors publishing solo, all 491 authors were involved in multi-author articles. An average of 11.49 citations/article was recorded during the study period. The scientific output related to *P*. *shigelloides* research by Lotka’s law showed a beta coefficient and constant of 2.30 and 0.44, respectively, with a Kolmogorov-Smirnoff goodness-of-fit of 0.92 (P = 0.46, two-sample t-test). The lack of a statistical difference between the theoretical and observed Lotka’s distributions indicates that Lotka’s law does not apply to *P*. *shigelloides* research productivity. Published studies on *P*. *shigelloides* from 1990 to 2017 and average total citations of articles by year are shown in Fig 1. The annual growth rate was −0.8%, with an overall mean of 5.54±2.66, suggesting that research on *P*. *shigelloides* has been decreasing overtime. Research output fluctuated during the survey period, peaking in 2000 and 2006 (each accounting for 7.7% [12/155] of the total). Similarly, average total citations of articles published fluctuated over the years and peaked in the year 2011 (average = 29.8).

**Table 1:** Summary information on retrieved *P*. *shigelloides* studies, 1990–2017.

Descriptions	Counts and rates
No. of articles	155
No. of authors	493
Involved in single-author articles	2
Involved in multi-author articles	491
Articles/author	0.31
Authors/article	3.18
Co-author appearances	673
Co-authors/article	4.34
Collaboration index (CI)	3.32
Average no. of citations/article	11.49
Study source (journals)	90
Keywords-Plus (ID)	398
Author’s keywords (DE)	259
Language	
English	147
German	4
Spanish	4

Table 2 shows the top 20 most productive authors in the field. K. Krovacek (Sweden) ranked first, co-authoring 12 (7.7%) articles; and I. Ciznar (Slovak Republic) was second with 11 (7.1%) articles. The h_index (total citations) was 7 (162) for K. Krovacek, and 6 (148) for I. Ciznar. It is worth noting that the topmost active authors were affiliated with institutions in developed nations, including Sweden (n = 6), USA (n = 5), Japan (n = 3), Spain (n = 5), Poland (n = 3), Czech Republic (n = 1), and Slovak Republic (n = 1).

**Table 2:** Top 20 productive authors on *P*. *shigelloides*.

Rank	Author	Affiliation	Nation	Articles	% of 155	h_index	TC
1	Krovacek, K.	Sveriges Lantbruksuniversitet Biomedical Centre	Sweden	12	7.7	7	162
2	Ciznar, I.	Institute of Preventive and Clinical Medicine	Slovak Republic	11	7.1	6	148
3	Aldova, E.	National Institute of Public Health Prague Czechia	Czech Republic	10	6.5	7	125
3	Lugowski, C.	Polish Academy of Sciences and University of Opole	Poland	10	6.5	7	142
4	Gonzalez-Rey, C.	Sveriges Lantbruksuniversitet, Biomedical Centre	Sweden	8	5.2	5	88
4	Lukasiewicz, J.	Polish Academy of Sciences	Poland	8	5.2	5	97
4	Niedziela, T.	Swedish University of Agricultural Sciences	Sweden	8	5.2	6	130
5	Levin, R.E.	University of Massachusetts	USA	7	4.5	3	24
6	Jachymek, W.	Swedish University of Agricultural Sciences	Sweden	6	3.9	6	123
6	Kaszowska, M.	Polish Academy of Sciences	Poland	6	3.9	3	29
6	Tomas, J.M.	Universidad de Barcelona	Spain	6	3.9	4	55
7	Gu, W.	University of Massachusetts	USA	5	3.2	2	16
7	Kenne, L.	Swedish University of Agricultural Sciences	Sweden	5	3.2	5	115
7	Merino, S.	Universidad de Barcelona	Spain	5	3.2	3	39
8	Henderson, D.P.	University of Texas at Austin	USA	4	2.6	4	67
8	Okawa, Y.	Tohoku Pharmaceutical University	Japan	4	2.6	4	53
8	Shimada, T.	National Institute of Infectious Diseases	Japan	4	2.6	3	36
8	Svenson, S.B.	Swedish University of Agricultural Sciences	Sweden	4	2.6	3	58
8	Tsugawa, H.	Tohoku Pharmaceutical University	Japan	4	2.6	4	53
9	*Aquilini, E.	University of Barcelona	Spain	3	1.9	2	12

Ranking based on the number of articles; TC, total citations.

*Shared with 9 others: Bravo, L. (Cuba), Corsaro, M.M. (Italy), Garcia-Lopez, M.L. (Spain), Hernandez, P. (Venezuela), Hostacka, A.(Slovakia), Lanzetta, R. (Italy), Obi, C.L. (Nigeria/South Africa), Otero, A. (Spain), Parrilli, M. (Italy), Pelayo, J.S. (Brazil), Pieretti, G. (Italy), Qadri, F. (Bangladesh), Sack, D.A. (USA), Santos, J.A. (Spain), Saridakis, H.O. (Brazil), Schneerson, R. (USA), Stock, I. (Germany),Yuen, K.Y. (China).

The 20 top cited articles on *P*. *shigelloides* are listed in S1 Table. These studies spanned the fields of infection, immunity, clinical microbiology, and biochemistry. The total no. of citations of the top-cited articles ranged from 35 to 111; most of these were the result of funded research.

Research output related to *P*. *shigelloides* for the top 20 most active countries is shown in Table 3. United States ranked first in terms of total number of articles (n = 29, 18.7%) and citations (n = 451), followed by Sweden (n = 14, 9.0%) and Germany (n = 10, 6.5%). The frequency of publication varied among the top countries from 1.3 to 18.7%. Sweden had highest productivity (1.563 article/million population) when normalized for population size using mid-period population (2003). The rank order of these countries changed when productivity was measured based on the number of citations per country, with only United States and Sweden remaining in the same positions. Other countries that made up the top 20 based on citations per country were Hong Kong (27.0), Australia and Finland (23.0–23.3). Asian countries in the top 20 list were Japan (n = 9), China (n = 7), Bangladesh (n = 5), and India (n = 3). Nigeria (n = 4) and Cameroon (n = 2) were the only African countries in the top 20 list.

**Table 3:** Most productive countries in terms of *P*. *shigelloides* research.

		Productivity based on no. of articles					Productivity based on no. of citations per country				
Rank	Country	Articles	SCP	MCP	Frequency (%)	A/MP	Rank	Country	TC	ACC	
1	USA	29	23	6	18.7	0.100	1	USA	451	15.6	
2	Sweden	14	1	13	9.0	1.563	2	Sweden	244	17.4	
3	Germany	10	9	1	6.5	0.121	3	Australia	93	23.3	
4	Japan	9	8	1	5.8	0.236	4	Japan	91	10.1	
4	Poland	9	5	4	5.8	0.071	5	Brazil	74	10.6	
5	Brazil	7	5	2	4.5	0.038	6	Germany	70	7.0	
5	China	7	6	1	4.5	0.005	7	England	68	17.0	
6	Czech Republic	6	5	1	3.9	0.589	8	Poland	62	6.9	
6	Spain	6	5	1	3.9	0.073	9	China	54	7.7	
7	Bangladesh	5	4	1	3.2	0.036	10	Czech Republic	50	8.3	
8	Australia	4	4	0	2.6	0.201	10	Spain	50	8.3	
8	England	4	3	1	2.6	0.070	11	France	49	16.3	
8	Italy	4	0	4	2.6	0.030	12	Italy	46	11.5	
8	Nigeria	4	4	0	2.6	0.001	13	Bangladesh	43	8.6	
9	France	3	2	1	1.9	0.558	14	Hong Kong	27	27.0	
9	India	3	3	0	1.9	0.116	15	Finland	23	23.0	
9	Slovakia	3	1	2	1.9	0.048	16	Canada	20	10.0	
9	Venezuela	3	3	0	1.9	0.003	17	Netherlands	16	16.0	
10*	Cameroon	2	2	0	1.3	0.121	18	Nigeria	15	3.8	
10*	Canada	2	0	2	1.3	0.063	19	India	13	4.3	

**SCP:** single country publications; **MCP:** multiple country publications; **A/MP:** Articles per million populations (2003 population); **TC:** Total Citations; **AAC:** Average Article Citations

*co-shared with Cuba, Switzerland and Turkey.

The top 20 journals with the most published articles on *P*. *shigelloides* are listed in S2 Table. These journals cover a range of subjects including carbohydrates, microbiology, food science, infectious disease, immunology, and biochemistry, reflecting active areas in *Plesiomonas* research. Carbohydrate Research ranked first (n = 9, 5.8%), followed by Journal of Clinical Microbiology, Folia Microbiologica and Food Biotechnology each with 6 articles (3.9%).

Table 4 shows the most relevant keywords related to *Plesiomonas* studies, including both author keywords (DE) and Keywords-Plus (ID). Both Author Keywords (DE) and Keywords-Plus (ID) have 10 keywords in common (lipopolysaccharide, *Aeromonas* species, antigens, oligosaccharide, disease, children, diarrhea, *Shigella*, virulence, and water). Fourteen keywords were unique to Author Keywords (*Plesiomonas shigelloides*, nuclear magnetic resonance (NMR), PCR, structure, MALDI-TOF, fish, pathogenicity, media, meningoencephalitis, resistance, enterotoxin, gastroenteritis, serotyping, and sepsis), and 13 keywords were unique to Keywords-Plus (infections, *Escherichia*, septicemia, environments, *in-vitro*, polysaccharide, *Vibrio* species, bacteria, biological repeating unit, iron, meningitis, humans, and strains). The unique Author Keywords primarily described medium of transmission (fish) and methods involved in isolation and characterization of the microorganism (NMR, MALDI-TOF, PCR, enterotoxin, resistance, pathogenicity, serotyping, and structure) and specific infections (meningoencephalitis, gastroenteritis, and sepsis). Author keyword terms associated with identification methods of *P*. *shigelloides* included polymerase chain reaction (PCR, n = 8, 5.2%), matrix-assisted laser desorption/ionization–time-of-flight mass spectrometry (MALDI-TOF, n = 6, 3.9%), and serotyping (n = 3, 1.9%). A total of 82 (52.9%) articles reported author keywords (DE) related to *Plesiomonas shigelloides*. Keywords in articles focusing on analysis of *P*. *shigelloides* cell wall structure included lipopolysaccharide (n = 13—, 8.4% (DE); n = 21, 13.6% (ID)), (NMR; n = 13, 8.4% (DE)), structure (n = 7, 4.5% (DE)), antigens (n = 6, 3.9% (DE), n = 19, 12.3% (ID)), biological repeating unit (n = 11, 7.1% (ID), and oligosaccharide (n = 6, 3.9, n = 11, 7.1% (ID)). The Keyword analysis identified diarrhea in 4 (2.6%) and 43 (27.7%) articles by author keyword and keyword pus respectively. Co-infection with *Escherichia coli* (n = 24, 15.5% (ID)) and *Aeromonas* species (n = 6, 3.9% (DE), n = 41, 26.5% (ID)) was represented. Keywords linked to extraintestinal *P*. *shigelloides* infection included septicemia (n = 24, 15.5% (ID)), meningoencephalitis (n = 4, 2.6 (DE)), Sepsis (n = 3, 1.9% (DE)), and meningitis (n = 10, 6.5% (ID)), which ranked 5th, 11th, and 11th respectively.

**Table 4:** Most relevant keywords.

Rank	Author keywords (DE)	Freq. (% of 155)	Rank	Keywords-Plus (ID)	Freq. (% of 155)
1	*Plesiomonas shigelloides*	82(52.9)	1	Diarrhea	43(27.7)
2	Lipopolysaccharide	13(8.4)	2	*Aeromonas* species	41(26.5)
2	NMR	13(8.4)	3	Infections	31(20.0)
3	PCR	8(5.2)	4	Escherichia	29(18.7)
4	Structure	7(4.5)	5	Septicemia	24(15.5)
5	*Aeromonas species*	6(3.9)	4	Lipopolysaccharide	21(13.6)
6	Antigens	6(3.9)	5	Disease	20(12.9)
6	MALDI-TOF	6(3.9)	5	Antigens	19(12.3)
7	Oligosaccharide	6(3.9)	6	Water	15(9.7)
8	Disease	5(3.2)	7	Environments	14(9.0)
9	Fish	5(3.2)	8	*In-vitro*	13(8.4)
11	Pathogenicity	5(3.2)	8	Polysaccharide	13(8.4)
11	Children	4(2.6)	9	*Vibrio* species	12(7.7)
11	Diarrhea	4(2.6)	10	Bacteria	11(7.1)
11	Media	4(2.6)	10	Biological repeating unit	11(7.1)
11	Meningoencephalitis	4(2.6)	10	Humans	11(7.1)
11	Resistance	4(2.6)	10	Oligosaccharide	11(7.1)
12	Enterotoxin	3(1.9)	10	*Shigella*	11(7.1)
12	Gastroenteritis	3(1.9)	11	Children	10(6.5)
12	Sepsis	3(1.9)	11	Iron	10(6.5)
12	Serotyping	3(1.9)	11	Meningitis	10(6.5)
12	*Shigella*	3(1.9)	11	Strains	10(6.5)
12	Virulence	3(1.9)	11	Virulence	10(6.5)
12	Water	3(1.9)			

MALDI–TOF MS, matrix-assisted laser desorption/ionization–time-of-flight mass spectrometry; NMR, nuclear magnetic resonance; PCR, polymerase chain reaction.

**Fig 2:**
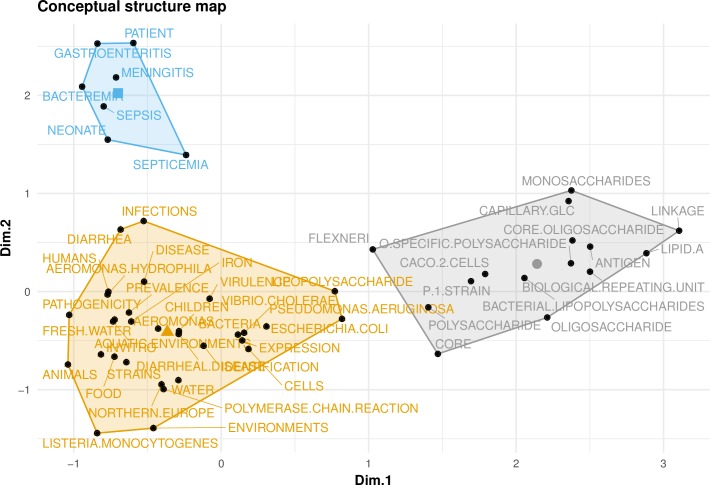
Common conceptual frames associated with *P*. *shigelloides* studies. The 155 retrieved articles showed K-means clustering with three clusters of sizes 8, 13, and 33 reflecting concepts frequently linked to *P*. *shigelloides*.

The common conceptual frames in retrieved articles determined by K-means clustering with three clusters of 8, 14, and 39 elements showed research responses focused on neonates (children) extraintestinal infections and gastroenteritis, elucidation of cell wall structure (lipopolysaccharides, core oligosaccharide, o-specific polysaccharide etc.), and co-infections of *P*. *shigelloides* with other pathogens, respectively (Fig 2). The 33-element cluster explained the co-occurrence and co-infection of *P*. *shigelloides* with other bacteria. Other indicators of frequently represented concepts and frameworks related to *P*. *shigelloides* included co-occurrence of terms and keywords. S1 Fig shows the co-occurrence network of the top 20 terms associated with *P*. *shigelloides* studies, while S2 Fig shows the co-occurrence networks of keywords. These concept-related frameworks or terms included virulence, meningoencephalitis, *Aeromonas*, newborn, antigen, pathogenicity, sepsis, diarrhea, infections, *Escherichia coli*, septicemia, biological repeating unit, diarrheal disease/gastroenteritis, lipopolysaccharide, water, iron, polysaccharide, bacteremia, meningitis, septicemia, lipid-A, strains, and aquatic environments.

**Fig 3:**
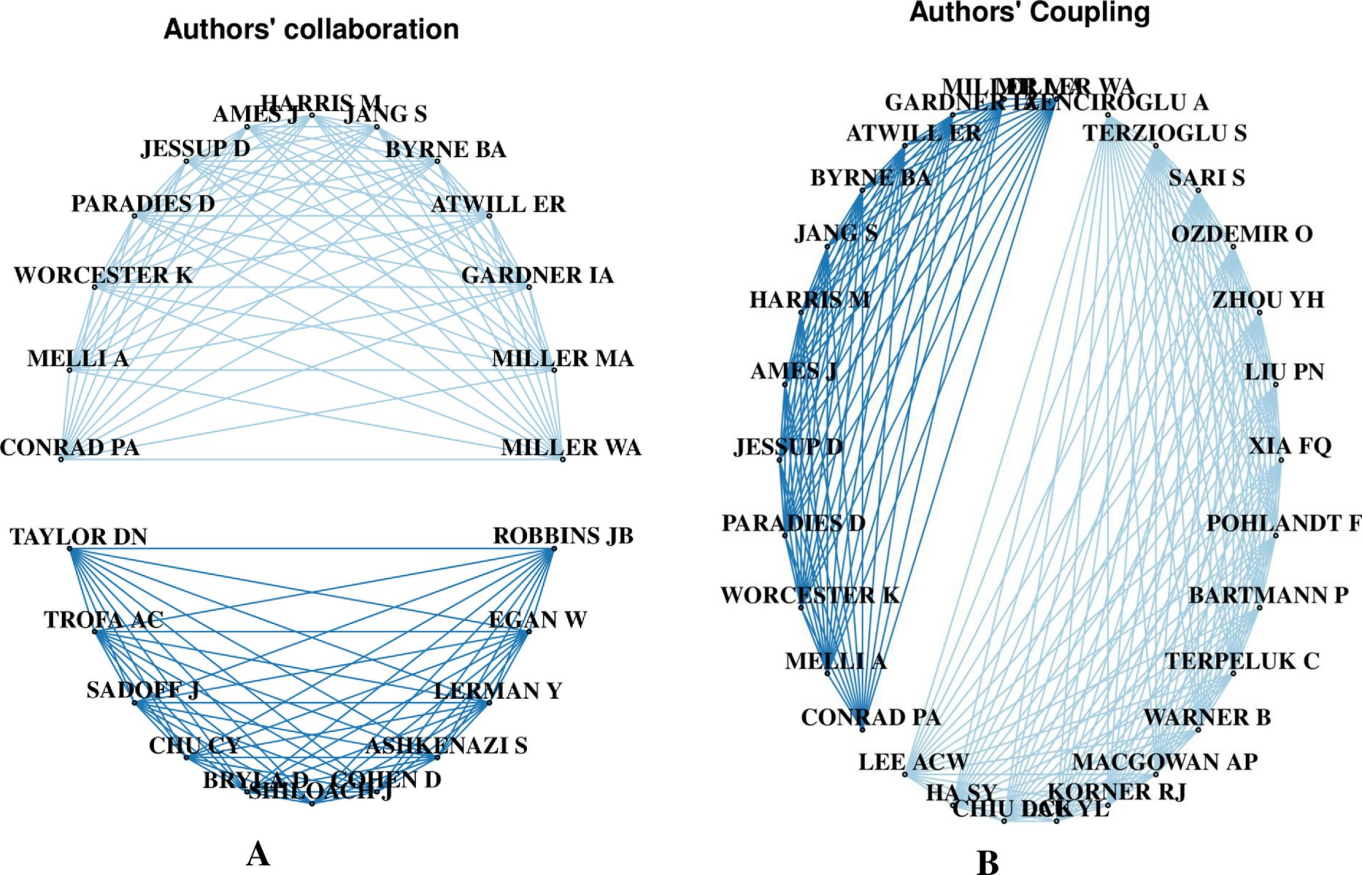
The top 20 authors’ collaboration and coupling networks on *P*. *shigelloides* studies. A. Top 20 authors’ collaboration networks on *P*. *shigelloides* studies. Each node in the network represents a different author’ collaboration with other authors. Connecting lines represent collaboration pathways between authors. The number of lines from a node corresponds to a number of co-authorship. B. Top 20 authors’ coupling networks on *P*. *shigelloides* studies. Each node in the network represents a different author coupling with other authors. Connecting lines represent coupling pathways between authors. The number of lines from a node corresponds to the number of articles that co-listed the author in their reference list.

The top 20 authors’ collaboration and coupling networks on *P*. *shigelloides* studies were divided into two mega-clusters or spheres with nodes occupied solely by researchers from high-income countries (Fig 3A and 3B). The first sphere of the authors’ network comprised 13 nodes (authors) with no fewer than 10 linkages, while the second sphere included 10 nodes (authors) with the number of collaboration linkages ranging from nine to 10. Similarly, the two separate authors’ coupling network spheres included 13 and 17 authors’ networks, respectively; collaborative conjugation ranged from eleven nodes (authors) in the former and 18 in the latter.

**Fig 4:**
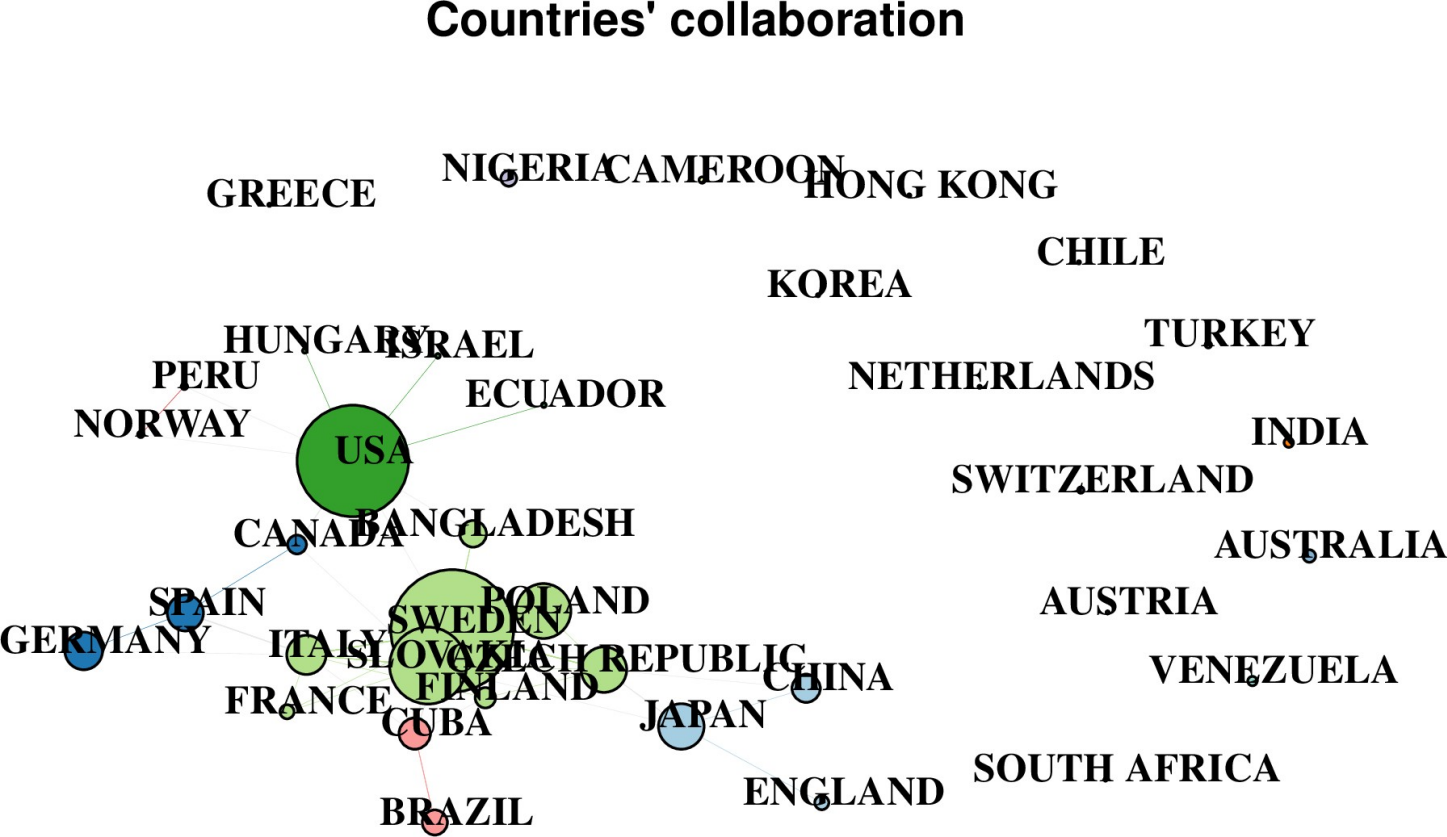
Fifty (50) countries’ collaboration networks on *P*. *shigelloides* studies. Each node in the network represents a different nation and the node’s diameter corresponds to the strength of a nation’s collaboration with other countries. Lines represent collaboration pathways between countries.

Fig 4 shows 50 countries’ collaboration networks on *P*. *shigelloides* studies. Collaboration pathways ranged from 1 to 9. The Sweden had a high number of collaborations (n = 9), followed by United States (n = 8), Slovakia (n = 8), and Italy (n = 7). Other countries had no collaboration networks. Prominent network color code: green, USA network; purple, Spain network; light green, Sweden network; pink, Cuba-Brazil network; blue, Japan-China network.

## Discussion

The present bibliometric analysis of *P*. *shigelloides* examined global research trends between 1990 and 2017 based on data retrieved from WoS. We found that the number of research articles on *P*. *shigelloides* increased non-linearly from 5 to 155 articles. However, a negative trend in rate of increase was noted (−0.8%) suggesting that research on *P*. *shigelloides* has not been of broad interest in the past 27 years, likely due to discontinuation of *Plesiomonas*-related research by certain authors and differences in regional distribution of the microorganism. Furthermore, the function estimates and goodness-of-fit indicated that scientific output on *P*. *shigelloides* does not follow Lotka’s law, suggesting that the number of articles related to *Plesiomonas* research will further decline in the future. In general, emerging and re-emerging bacterial pathogens are not accorded the same degree of attention as their viral counterparts. Health emergencies (e.g., outbreaks of infection) relating to emerging viral pathogens including Zika and Chikungunya viruses have driven the generation of new scientific knowledge, resulting in a significant increase in the number of research articles on these subjects [68]. For instance, in 2005 only eight articles were published on Chikungunya virus, but by 2014, the number had reached 302 [69]. Similarly, only 43 articles on Ebola virus were published in 2013 prior to the Ebola outbreaks in West Africa, but this increased to more than 600 articles in 2014 [70].

As is the case with other research areas, most of the leading authors in the *Plesiomonas* research field were from developed nations such as the United States, Sweden, Austria, Japan, Spain, Poland, Czech Republic, and the Slovak Republic, with few from low-income countries, thus follow the similar trend of low productivity of the region in other research areas. It has been suggested that the economic strength (growth) of a nation influences the research output [43,71–73]. The higher prevalence of *Plesiomonas* infections in developing countries [74] should motivate researchers in countries most affected to carry out more studies on this pathogen.

Some of the most frequently cited studies pertained to health and identification of the pathogen—for example, efforts to produce a multivalent vaccine using O-specific polysaccharides from *Shigella* spp. and *P*. *shigelloides*; iron uptake in *Plesiomonas shigelloides*; and comparative analysis of *P*. *shigelloides* and *E*. *coli* (*Shigella*) *sonnei* O-antigen gene clusters. Others focused on *P*. *shigelloides* hemolytic expression; serological analysis of the *Plesiomonas* core; and multilocus sequence typing of *Plesiomonas* and its pathogenic potential. The United States and Sweden dominated the list of top 20 countries most actively researching *Plesiomonas* in terms of numbers of articles and citations. In addition to economic strength and availability of research facilities and funding [43,71–73], this productivity can be ascribed to a high level of intra-national and possibly multinational collaboration with other institutions, which can impact research visibility and citation frequency [43,73,75]. In particular, the dominance of the United States has been noted in other fields of research [42,54,76,77]. Also, authors’ multiple affiliations influence country collaboration network. Conversely, the low contributions from developing countries including countries from Africa characterized by a high frequency of self-funded or independent studies [78], mirror the situation of research in other fields. The shift in rank among the top 20 nations most active in the *Plesiomonas* research field when productivity was measured based on total citation per country ought not be regarded as a precise measure of productivity. Citation rate does not reflect publication output of an author or country [76], since the smaller the number of articles used for estimation, the larger the impact of a few frequently cited articles [76]. Self-citations and inaccurate citations can also provide false quality metrics [76].

The most frequently mentioned keywords and research areas (including publication outlets) associated with *Plesiomonas* studies reflect the research hotspot during the survey period, which included cell wall (Carbohydrate research), co-infection, and extraintestinal infections such as septicemia, bacteremia, and meningitis. These findings reveal the most pressing health issues related to *Plesiomonas*-induced gastroenteritis and extraintestinal infections, and co-infections with other pathogens and effort to gain an understanding of the structural architect of the pathogen’s cellwall; this was supported by other conceptual framework indicators such as co-words or keyword co-occurrence networks. However, important topics such as strain-based delineation and identification, including detection of pathogenic and non-pathogenic strains, that are necessary for infection management were lacking and were not evident from the bibliometric analyses. Newer research themes such as molecular and genomic-level studies as an alternative or complementary to traditional experimentation (which has some limitations) necessary to clarify the pathogenesis of *Plesiomonas* infection were not apparent throughout this study. A bibliometric survey complemented with a narrative review or meta-analysis may be beneficial in *Plesiomonas* research. Future research is needed to answer questions related to what particular strain of the microorganisms are pathogenic and how to differentiate pathogenic variants from non-pathogenic ones.

The two mega-clusters or spheres in the top authors’ collaboration and coupling networks on *P*. *shigelloides* studies showed collaboration pathways mainly among authors from high-income countries, which is similar to trends observed in collaboration network analyses of human immunodeficiency virus and human papilloma virus studies [78]. Alliances between developing and developed countries are rare in a number of scientific areas [78]. Among researchers in the United States, collaboration pathways were largely intra-national, as suggested by a large number of publications but 6 multiple country publications. In contrast, collaborations by authors in Cuba, Finland, Czech Republic, Italy, Slovakia, and Sweden tended to be multi-national, which is more valuable for the epidemiological control of pathogens. The absence of collaboration pathway in Venezuela, India, Nigeria, and other African nations is consistent with the low number of publications from these countries. Intra- and international collaborations between developed and developing nations could provide opportunities for the division of labor and resources to address important scientific questions.

There were some limitations related to the bibliometric survey adopted in this study, including the use of a single database (the WoS), the low sensitivity and strictness of the search terms and search strategy used, and the exclusions of other document types (e.g., meeting abstract, note and proceeding papers etc.) and articles published in non-English Language (e.g in Chinese). Also, the current analysis did not allow for a narrative review and judgment on contents and results of the articles [79]. As noted earlier, emerging themes and recent research focus in *Plesiomonas* are not easily recognized in bibliometric studies due to low frequency of appearance in keywords. Notwithstanding these limitations, this is the first bibliometric study on Plesiomonas-related research contributing to the evidence base and would help direct future research. Also, the WoS has a larger coverage compared to other database, reliable indexing technology that minimizes the ‘‘indexer effect” and is well accepted among scientific communities [80].

## Conclusion

Our bibliometric analysis revealed a global diminishing research in *Plesiomonas*, greater research output from high-income countries compared to low- and middle-income countries and limited collaboration with developing countries. The low productivity in developing countries in *Plesiomonas* research mirror the state of affairs in other research fields. A better understanding of the clinical features, epidemiology and *Plesiomonas*-associated diseases is needed in countries with high infection rates. Emerging themes and recent research focus in *Plesiomonas* research are not easily recognized in bibliometric studies due to low frequency of appearance in keywords and, hence, the need for future studies guided by narrative reviews.

## Supporting information

S1 Fig Co-occurrence network of top 20 terms associated with *P*. *shigelloides* studies. Each node in the network represents a different term. The node’s diameter corresponds to the frequency of co-occurrence with other terms. Lines depict co-occurrence pathways between terms.
(TIF)

S2 Fig Keyword co-occurrence networks related to *P*. *shigelloides* studies. Each node in the network represents one of the top 20 keywords. The node’s diameter corresponds to the keyword’s frequency of co-occurrence with other keywords. Lines depict co-occurrence pathways between keywords.
(TIF)

S1 Table Top 20 studies per citation (most frequently cited manuscripts). (DOCX)

S2 Table Top 20 journals with the most published articles on *P*. *shigelloides*. (DOCX)

S1 Appendix The detailed search Boolean for articles identification from WoS. (DOCX)
