# Supplementary figures and images for: A global bibliometric analysis of Plesiomonas-related research (1990 – 2017)
Source: PLoS One. 2018 Nov 29;13(11):e0207655. doi: 10.1371/journal.pone.0207655 (PMC6264487; doi:10.1371/journal.pone.0207655)

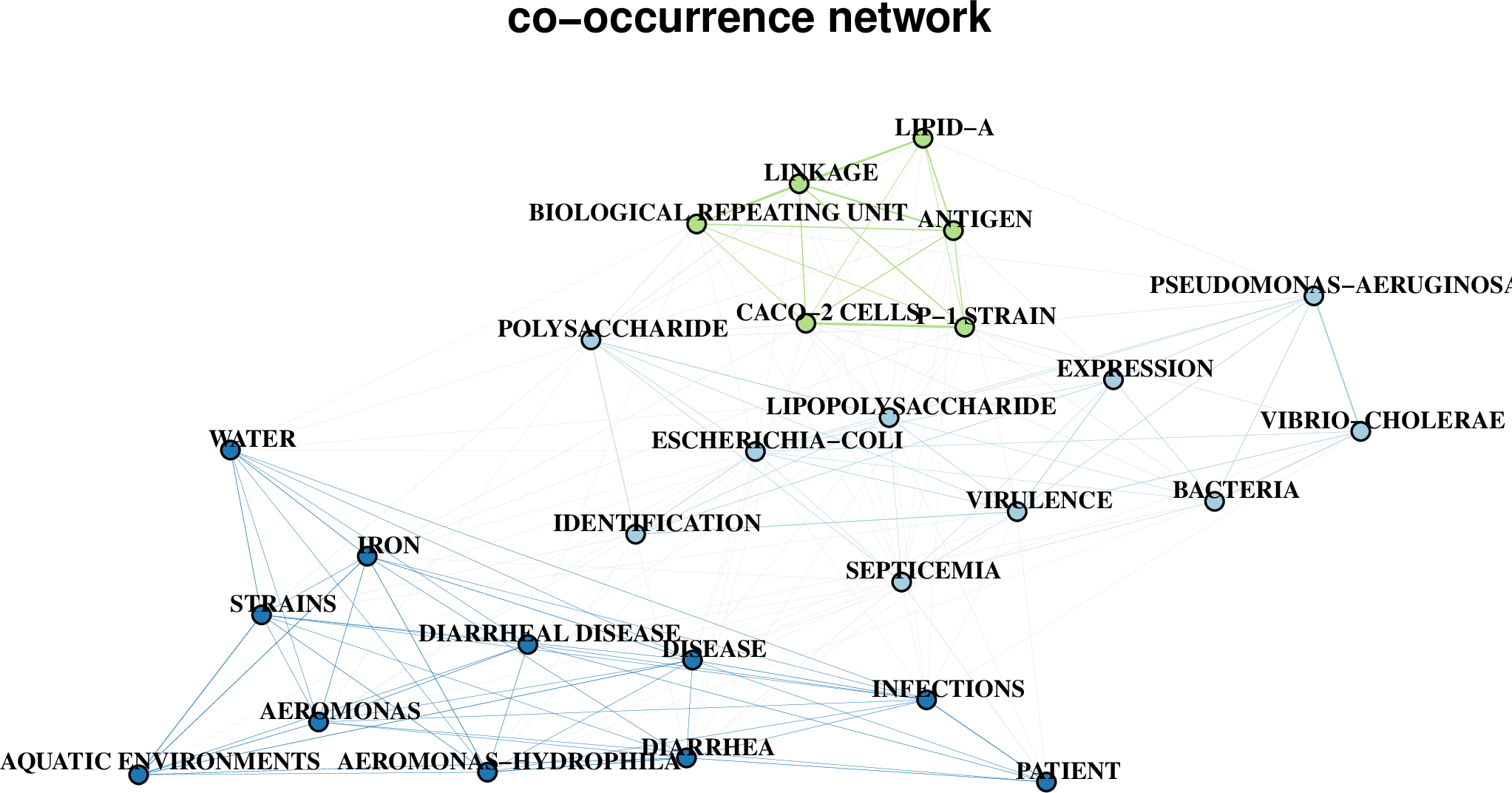

Supplement: S1 Fig — Each node in the network represents a different term. The node’s diameter corresponds to the frequency of co-occurrence with other terms. Lines depict co-occurrence pathways between terms. (TIF) [file pone.0207655.s001.tif]

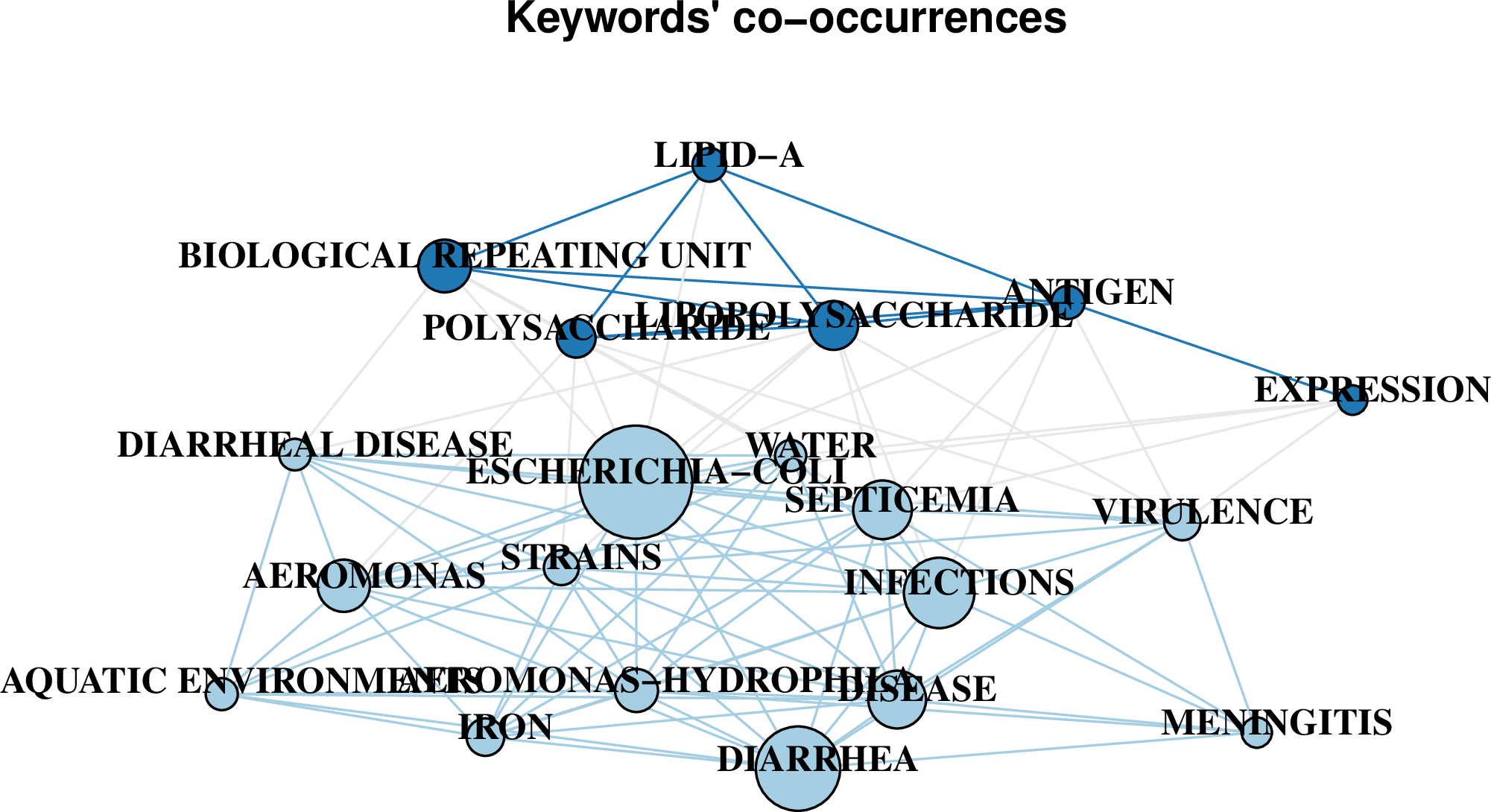

Supplement: S2 Fig — Each node in the network represents one of the top 20 keywords. The node’s diameter corresponds to the keyword’s frequency of co-occurrence with other keywords. Lines depict co-occurrence pathways between keywords. (TIF) [file pone.0207655.s002.tif]
