# Supplementary material for: A global bibliometric analysis of Plesiomonas-related research (1990 – 2017)
Source: PLoS One. 2018 Nov 29;13(11):e0207655. doi: 10.1371/journal.pone.0207655 (PMC6264487; doi:10.1371/journal.pone.0207655)
Supplement: S1 Table — (DOCX) [file pone.0207655.s003.docx]

**S1 Table.** Top 20 studies per citation (most frequently cited manuscripts)

| **Rank** | **Author (year), journal** | **Title** | **Document** | **TC** | **TC/Year** | **Funding report** |
| --- | --- | --- | --- | --- | --- | --- |
| 1 | Taylor et al. (1993),  Infect. Immun. | Synthesis, characterization, and clinical evaluation of conjugate vaccines composed of the O-specific polysaccharides of *Shigella dysenteriae* type 1, *Shigella flexneri* type 2a, and *Shigella sonnei* (*Plesiomonas shigelloides*) bound to bacterial toxoids | Article | 111 | 4.440 | Not reported |
| 2 | Daskaleros et al. (1991),  Infect. Immun. | Iron uptake in *Plesiomonas shigelloides*: cloning of the genes for the heme-iron uptake system | Article | 72 | 2.667 | Funded |
| 3 | Shepherd et al. (2000),  Infect. Immun. | Comparison of O-antigen gene clusters of *Escherichia coli* (Shigella) sonnei and *Plesiomonas shigelloides* O17: sonnei gained its current plasmid-borne O-antigen genes from *P. shigelloides* in a recent event | Article | 64 | 3.556 | Funded |
| 4 | Janda and Abbott (1993),  J. Clin. Microbiol. | Expression of hemolytic activity by *Plesiomonas shigelloides* | Article | 44 | 1.760 | Not reported |
| 5 | Salerno et al. (2007), J. Bacteriol. | Recombining population structure of *Plesiomonas shigelloides* (Enterobacteriaceae) revealed by multilocus sequence typing | Article | 43 | 3.909 | Funded |
| 6 | Niedziela et al. (2002),  J. Biol. Chem. | Core oligosaccharides of *Plesiomonas shigelloides*O54: H2 (Strain CNCTC 113/92) structural and serological analysis of the lipopolysaccharide core region, the O-antigen biological repeating unit, and the linkage between them | article | 40 | 2.500 | Funded |
| 7 | Czaja et al. (2000),  Eur. J. Biochem. | Structural studies of the O-specific polysaccharide from *Plesiomonas shigelloides* strain CNCTC 113/92 | Article | 40 | 2.222 | Funded |
| 8 | Miller et al. (2006),  Microb. Ecol | *Salmonella* spp., *Vibrio* spp., *Clostridium perfringens*, and *Plesiomonas shigelloides* in marine and freshwater invertebrates from coastal California ecosystems | Article | 39 | 3.250 | Funded |
| 9 | Henderson et al. (2001),  J. Bacteriol. | Characterization of the *Plesiomonas shigelloides* genes encoding the heme iron utilization system | Article | 36 | 2.118 | Funded |
| 10 | Abbott et al. (1991),  J. Clin. Microbiol. | Laboratory investigations on the low pathogenic potential of *Plesiomonas shigelloides* | Article | 35 | 1.296 | Not reported |
| 11 | Krovacek et al. (2000), Comp. Immunol. Microbiol. Infect. Dis. | Isolation, biochemical and serological characterisation of *Plesiomonas shigelloides* from freshwater in Northern Europe | Article | 28 | 1.556 | Funded |
| 12 | Lee et al. (1996),  Pediatr. Hematol. Oncol. | *Plesiomonas shigelloides* septicemia: Case report and literature review | Article | 27 | 1.227 | Not reported |
| 13 | Gonzalez-Rey et al. (2000), Fems Immunol. Med. Microbiol. | Specific detection of *Plesiomonas shigelloides* isolated from aquatic environments, animals and human diarrhoeal cases by PCR based on 23S rRNA gene | Article | 25 | 1.389 | Not reported |
| 14 | Jiang et al. (1991),  J. Infect. Dis. | Intestinal secretory immune-response to infection with *Aeromonas* species and *Plesiomonas shigelloides* among students from the United-States in Mexico | Article | 25 | 0.926 | Not reported |
| 15 | Kowal et al. (2002), Biochemistry | New UDP-GlcNAc C4 epimerase involved in the biosynthesis of 2-acetamino-2-deoxy-L-altruronic acid in the O-antigen repeating units  of *Plesiomonas shigelloides* O17 | Article | 24 | 1.500 | Not reported |
| 16 | Olsvik et al. (1990),  J. Clin. Microbiol. | Laboratory observations on *Plesiomonas shigelloides* strains isolated from children with diarrhea in peru | Article | 24 | 0.857 | Not reported |
| 17 | Baratela et al. (2001),  J. Appl. Microbiol. | Effects of medium composition, calcium, iron and oxygen on haemolysin production by *Plesiomonas shigelloides* isolated from water | Article | 23 | 1.353 | Not reported |
| 18 | Shigematsu et al. (2000), Epidemiol. Infect. | An epidemiological study of *Plesiomonas shigelloides* diarrhoea among Japanese travellers | Article | 23 | 1.278 | Not reported |
| 19 | Rautelin et al. (1995), Scand. J. Infect. Dis. | Enteric *Plesiomonas shigelloides* infections in finnish patients | Article | 23 | 1.000 | Not reported |
| 20 | Okawa et al. (2004),  Fems Microbiol. Lett. | Isolation and characterization of a cytotoxin produced by *Plesiomonas shigelloides* P-1 strain | Article | 22 | 1.571 | Not reported |

TC, total citations.
