# Supplementary material for: A global bibliometric analysis of Plesiomonas-related research (1990 – 2017)
Source: PLoS One. 2018 Nov 29;13(11):e0207655. doi: 10.1371/journal.pone.0207655 (PMC6264487; doi:10.1371/journal.pone.0207655)
Supplement: S2 Table — (DOCX) [file pone.0207655.s004.docx]

**S2 Table.** **Top 20 journals with the most published articles on *P. shigelloides***

| **Rank** | **Sources** | **Articles** | **% of 155** |
| --- | --- | --- | --- |
| 1 | Carbohydrate Research | 9 | 5.81 |
| 2 | Folia Microbiologica | 6 | 3.87 |
| 3 | Food Biotechnology | 6 | 3.87 |
| 4 | Journal of Clinical Microbiology | 6 | 3.87 |
| 5 | Infection and Immunity | 4 | 2.58 |
| 6 | Journal of Applied Microbiology | 4 | 2.58 |
| 7 | Journal of Food Protection | 4 | 2.58 |
| 8 | Journal of Medical Microbiology | 4 | 2.58 |
| 9 | Scandinavian Journal of Infectious Diseases | 4 | 2.58 |
| 10 | Comparative Immunology Microbiology And Infectious Diseases | 3 | 1.94 |
| 11 | European Journal of Biochemistry | 3 | 1.94 |
| 12 | European Journal of Organic Chemistry | 3 | 1.94 |
| 13 | FEMS Microbiology Letters | 3 | 1.94 |
| 14 | Journal of Bacteriology | 3 | 1.94 |
| 15 | Veterinarni Medicina | 3 | 1.94 |
| 16 | African Journal of Biotechnology | 2 | 1.29 |
| 17 | Applied And Environmental Microbiology | 2 | 1.29 |
| 18 | Archiv Fur Lebensmittelhygiene | 2 | 1.29 |
| 19 | Archivos Latinoamericanos De Nutricion | 2 | 1.29 |
| 20 | East African Medical Journal | 2 | 1.29 |
