# Supplementary material for: A global bibliometric analysis of Plesiomonas-related research (1990 – 2017)
Source: PLoS One. 2018 Nov 29;13(11):e0207655. doi: 10.1371/journal.pone.0207655 (PMC6264487; doi:10.1371/journal.pone.0207655)
Supplement: S1 Appendix — (DOCX) [file pone.0207655.s005.docx]

**S1 Appendix. The detailed search Boolean for articles identification from WoS**

Title ("*Plesiomonas shigelloides*")

DOCUMENT TYPES: research article excluding (LETTER OR CORRECTION OR CORRECTION ADDITION OR NOTE OR PROCEEDINGS PAPER OR EDITORIAL MATERIAL OR MEETING ABSTRACT OR REVIEW OR BOOK CHAPTER)

Timespan: 1990-2017.

Indexes: SCI-EXPANDED, SSCI, A&HCI, CPCI-S, CPCI-SSH, BKCI-S, BKCI-SSH, ESCI, CCR-EXPANDED, IC.

TI=("*Plesiomonas shigelloides*") AND DT2= article AND NOT (LETTER OR CORRECTION OR CORRECTION ADDITION OR NOTE OR PROCEEDINGS PAPER OR EDITORIAL MATERIAL OR MEETING ABSTRACT OR REVIEW OR BOOK CHAPTER )

Timespan: 1990-2017. Indexes: SCI-EXPANDED, SSCI, A&HCI, CPCI-S, CPCI-SSH, BKCI-S, BKCI-SSH, ESCI, CCR-EXPANDED, IC.
